# Targeting HOXA11‐AS to mitigate prostate cancer via the glycolytic metabolism: In vitro and in vivo

**DOI:** 10.1111/jcmm.18227

**Published:** 2024-03-23

**Authors:** Jiankang Zhang, Sailian Li, Mengyu Zhang, Zhenting Wang, Zengshu Xing

**Affiliations:** ^1^ Department of Urology Affiliated Haikou Hospital of Xiangya Medical School, Central South University Haikou China; ^2^ Department of Gastroenterology Affiliated Haikou Hospital of Xiangya Medical School, Central South University Haikou China

**Keywords:** diagnostic model, epithelial‐mesenchymal transition, glycolytic metabolism, lncRNA, prostate cancer

## Abstract

As oncogenes or oncogene suppressors, long‐stranded non‐coding RNAs are essential for the formation and progression of human tumours. However, the mechanisms behind the regulatory role of RNA HOXA11‐AS in prostate cancer (PCa) are unclear. PCa is a common malignant tumour worldwide, and an increasing number of studies have focused on its metabolic profile. Studies have shown that the long non‐coding RNA (lncRNA) HOXA11‐AS is aberrantly expressed in many tumours. However, the role of HOXA11‐AS in PCa is unclear. This work aimed to determine how HOXA11‐AS regulated PCa in vitro and in vivo. We first explored the clinical role of HOXA11‐AS in PCa using bioinformatics methods, including single sample gene set enrichment analysis (ssGSEA), weighted gene co‐expression network analysis (WGCNA), and least absolute shrinkage and selection operator (LASSO)‐logistics systematically. In this study, PCa cell lines were selected to assess the PCa regulatory role of HOXA11‐AS overexpression versus silencing in vitro, and tumour xenografts were performed in nude mice to assess tumour suppression by HOXA11‐AS silencing in vivo. HOXA11‐AS expression was significantly correlated with clinicopathological factors, epithelial‐mesenchymal transition (EMT) and glycolysis. Moreover, key genes downstream of HOXA11‐AS exhibited good clinical diagnostic properties for PCa. Furthermore, we studied both in vitro and in vivo effects of HOXA11‐AS expression on PCa. Overexpression of HOXA11‐AS increased PCa cell proliferation, migration and EMT, while silencing HOXA11‐AS had the opposite effect on PCa cells. In addition, multiple metabolites were downregulated by silencing HOXA11‐AS via the glycolytic pathway. HOXA11‐AS silencing significantly inhibited tumour development in vivo. In summary, silencing HOXA11‐AS can inhibit PCa by regulating glucose metabolism and may provide a future guidance for the treatment of PCa.

## 1 INTRODUCTION

Prostate cancer (PCa) is one of the most frequently occurring forms of cancer among males globally.^1^ Most PCa patients have a 5‐year survival rate of almost 100%. However, the mortality rate for PCa patients with distant metastases is only 28%. As a result, PCa is one of the deadliest cancers.^2^ A previous study has shown that many lncRNAs were dysregulated in PCa.^3^ RNA transcripts with a length of more than 200 nucleotides that are unable to code for proteins are known as long‐stranded non‐coding RNAs (lncRNAs).^4^ LncRNAs play a critical role in various biological processes, including tumorigenesis.^5^ Growing evidence has shown that serum lncRNAs serve as biomarkers for various types of cancer.^6^ The *homology box (HOX)* gene cluster constitutes one of the most highly conserved polygenic loci in eukaryotes. The homologous HOX protein is a crucial component of the main regulatory system in the body.^7^ HOXA11‐AS can trigger tumour formation and metastasis, and act as an oncogene in various cancers by opening large chromatin structural domains, maintaining the state of chromatin or regulating RNA interference‐mediated silencing to promote epigenetic activation and repression.^8^, ^9^ HOXA11‐AS may act as a useful biomarker for the diagnosis and prognosis prediction of cancer patients.^10^

Epithelial‐mesenchymal transitions (EMTs) play a critical role in PCa development. Most deaths from PCa result from metastasis, which is associated with EMT, in which the characteristics of epithelial cells are transformed to result in the mesenchymal phenotype.^11^ EMT is closely related to the invasion and metastasis of malignant tumours such as those occurring in PCa patients. A previous study has shown that lncRNAs regulated tumour invasion and metastasis through EMTs.^12^ Previous studies have shown that E‐cadherin, N‐cadherin and vimentin expression levels might be affected by the regulation of HOXA11‐AS expression in other cancers.^13^, ^14^ However, the mechanism underlying the regulation of EMT by HOXA11‐AS in PCa is unclear.

Energy metabolism reprogramming induced by the EMT process is considered a key event in PCa. Aerobic glycolysis is one of the most prominent aspects of this metabolic reprogramming process.^15^ Instead of mitochondrial oxidative phosphorylation, cancer cells adopt the glucose‐dependent glycolytic pathway to generate energy, even when oxygen is present, specifically via the Warburg effect.^15^ Enhancing the Warburg effect is key to tumour growth and results in poor prognosis prediction for cancer patients. Abnormal metabolic changes are essential for tumour progression and promotion of cancer cell proliferation by boosting energy production and preserving redox balance. Several processes, including the metabolism of lipids, amino acids and glucose, are involved in the tumour metabolism process.

Consequently, the prevention of metabolic imbalances can reduce tumour cell growth. Studies have demonstrated the role of aerobic glycolysis in promoting PCa progression. For example, the PRKAR2B‐HIF‐1α loop enhances the Warburg effect and results in a growth advantage to PCa cells.^15^ Pten−/p53‐deficiency‐driven PCa tumour growth requires HK2‐regulated aerobic glycolysis. Furthermore, circ_0057553 promotes aerobic glycolysis and malignant behaviour in PCa cells by targeting YES1.^16^ Therefore, it is also necessary to investigate how HOXA11‐AS affects aerobic glycolysis in PCa cells.

The loss of epithelial markers is an important process in cells undergoing EMTs that is regulated by important transcriptional repressors.^17^ Our earlier study revealed that HOXA11‐AS is abundantly expressed in PCa and encourages the proliferation and migration of PCa cells.^18^ However, the mechanism by which HOXA11‐AS promotes EMT in PCa has not been clarified. In this study, HOXA11‐AS was used as the target in a combination of bioinformatics approaches, to explore the molecular mechanism of HOXA11‐AS and the promotion of EMTs in PCa at the cellular and global levels, respectively, to elucidate the prognostic and clinicopathological significance of lncRNA HOXA11‐AS expression in cancer patients and to identify new theoretical and therapeutic targets for PCa metastasis. In order to identify novel theoretical and therapeutic targets for PCa metastasis, it is important to understand the predictive and clinicopathological importance of lncRNA HOXA11‐AS expression in individuals with cancer.

## 2 MATERIALS AND METHODS

### 2.1 Acquisition of EMT and glycolytic gene sets

We get clinical follow‐up data as well as TCGA‐PRAD gene expression level data (all normalized log_2_ (FPKM+1) expression level data) from the TCGA database (https://gdc‐portal.nci.nih.gov/). It contains 52 normal samples and 499 PCa samples. Also, the sequencing platform was downloaded from the NCBI GEO (https://www.ncbi.nlm.nih.gov). The NCBI GEO database was used to download the GSE32571 sequencing platform GPL6947 Illumina HumanHT‐12 V3.0, containing 39 normal samples and 59 PCa samples.

Expression data related to HOXA11‐AS were extracted, and the diagnostic performance of HOXA11‐AS was assessed using the diagnostic ROC curve by comparing its differential profile between PCa versus normal by the Wilcoxon test and comparing its differential expression in different clinical stages.

From MSigDB v7.4 with the keywords “HALLMARK_EPITHELIAL_MESENCHYMAL_TRANSITION and HALLMARK_ glycolysis” as keywords and retrieved the related EMT and glycolysis pathway gene sets, containing 200 EMT‐related genes and 200 glycolysis‐related genes, respectively. In addition, 1011 EMT‐associated genes were downloaded from the dbEMT 2.0 database, and then all EMT and glycolysis‐associated genes were combined and extracted from the set for subsequent analysis.

### 2.2 HOXA11‐AS correlation analysis with EMT and glycolysis

Based on the hallmark_EMT and hallmark_glycolysis gene sets were estimated by the ssGSEA algorithm for EMT and glycolysis scores. The enrichment differences between the EMT and glycolysis scores in PCa versus normal were compared using the Wilcoxon test. Finally, the HOXA11‐AS was calculated by the cor function with the correlation between EMT glycolysis scores.

### 2.3 Construction and validation of the diagnostic model

Weighted gene co‐expression network analysis (WGCNA) determined gene clusters strongly relevant to the phenotypes of the samples to summarize the modular feature genes in these clusters. There are definitions for the neighbourhood function, scale‐free gene network and gene co‐expression correlation matrix. The gene modules associated with particular phenotypes were found by the dissimilarity coefficients of various nodes.^19^ We screened the gene modules connected to HOXA11‐AS as a trait for further investigation using the concatenated gene expression data found by 1.3 using the R program WGCNA.

Based on the modular genes identified previously, diagnostic genes with *p* < 0.05 were screened using one‐way logistic regression analysis. The LASSO‐Logistic algorithm (parameter selection: min a range of variance to obtain the lambda value of the simplest model, 1se gives a model with good performance and a minimum number of independent variables) in R3.6.1 language lars package was then used to filter the optimal model genes by 10‐fold cross‐validation with penalty parameter adjustment. The following diagnostic score formula was developed using the regression coefficients of each gene and the expression levels of the model genes.

$$\text{Diagnostic}\_\text{score}=\sum_{i=1}^{n}\text{coef}i\times \mathrm{EXP}i$$

In this formula, each gene's expression value is denoted by the letters EXP and coef, respectively.

Diagnostic scores were then compared by the Wilcoxon test for differences in PCa versus normal samples, using the TCGA training dataset and independent validation dataset through the ROC curve. The diagnostic models was assessed in GSE32571.

### 2.4 Cell culture

PCa cells DU145 and 22Rv1 and prostate epithelial cells (RWPE‐1) were obtained from Shanghai Cell Bank (Shanghai, China). DMEM (HyClone, USA) media supplemented with 10% FBS (Bioindustries, ISR) and 1% penicillin–streptomycin (Invitrogen) was used to culture the cells. Cells were grown in a 5% CO_2_ atmosphere at 37°C.

### 2.5 Cell transfection

Gene overexpression (OE‐HOXA11‐AS) and control (OE‐Con) plasmids were purchased from GenePharma (Shanghai, China). Small interfering RNAs for the HOXA11‐AS silencing group (si‐HOXA11‐AS) and the control group (si‐Con) were designed, synthesized and validated by GenePharma (Shanghai, China). All these plasmids and oligonucleotides were transfected into cells according to the Lipofactomine 3000 liposome transfection instructions following standard procedures.

### 2.6 Cell proliferation and invasion analysis

A total of 5 × 10^3^ DU145 or 22Rv1 per well were placed into the 48‐well plates. Cells were treated with HOXA11‐AS overexpression. Cell viability was monitored with MTT (Methylthiazolyldiphenyl‐tetrazolium bromide) (ST316, Beyotime, China). MTT reagent was mixed and incubated with cells for 2–3 h. Sample absorbance was recorded at 490 nm at the same time every day for three consecutive days with a microplate reader (Thermo, USA). Proliferation inhibition rate = [(average OD value of control group cells − average OD value of experimental group cells)/average OD value of control group cells] × 100%. Proliferation curves of respective treatment groups were plotted. Cell invasion assays were conducted using 8 μm Matrigel invasion chambers (Corning, USA) following a 48‐h transfection treatment. Following fixation, air‐drying, and staining with 1% crystal violet, images were acquired using a microscope.

### 2.7 Quantitative real‐time PCR

Total RNA was extracted using the RNA High Purity Total RNA Rapid Extraction Kit (Spi‐column) (Bio Teke Corporation, Beijing, China) in order to measure the expression level of HOXA11‐AS. The total RNA concentration was determined and reverse‐transcribed using the RT‐First Strand cDNA Synthesis Kit (Thermo, K1622). The primers were produced by Invitrogen and created by NCBI Primer Blast. The 2‐ct approach was used to analyse relative gene expression.

### 2.8 Western blotting

Total protein were produced using the RIPA protein extraction reagent (Beyotime, Beijing, China) in accordance with the manufacturer's instructions. To ascertain the concentrations, the Enhanced BCA Protein Assay Kit (Beyotime, Beijing, China) was used. Transferred on a PVDF membrane after being separated on a 10% SDS‐PAGE gel. The membranes were sealed with 5% skimmed milk for 1 h, followed by an overnight incubation at 4°C with the primary antibody against GAPDH, MMP‐2, MMP‐9, N‐cadherin, E‐cadherin and Vimentin. The membranes were cleaned using TBS buffer (Sigma Aldrich, St. Louis, MO, United States) and subjected to incubation of HRP‐linked secondary antibody at room temperature. The ECL reagent was used to observe the results (Thermo Fisher Scientific). Finally, we used ImageJ to assess the band density.

### 2.9 Wound healing assay

In 6‐well plates, DU145 and 22RV1 cells were injected and allowed to increase in density to 70%. Using a 200 L pipette tip that is sterile, the surface was scratched. The wells containing DMSO were filled with new media containing 2% fetal bovine serum. After 48 h of treatment, the well plates were imaged using a fluorescent microscope, 4% PFA was applied to the wells for fixation, and the area occupied by migrating cells was calculated.

### 2.10 Clone formation assay

We assessed cell colonies in the control, lncRNA overexpression, and lncRNA silencing groups. Logarithmic growth phase cells were taken and inoculated in 6‐well plates at 37°C and 5% CO_2_ at 500 cells/well. After a 14‐day culture, cell colonies were fixed at room temperature. The gorse colonies were then manually counted after the cell colonies were dyed with 0.1% crystal violet for 5 min and then photographed.

### 2.11 Gas chromatography–mass spectrometry analysis

Gas chromatography–mass spectrometry (GC–MS) was used to compare the metabolite differences between unmodified and HOXA11‐AS silenced DU145 cells. Culture dishes were applied to grow DU145 cells. After two PBS solution washes, the cells were scraped and stripped. The cells that had precipitated were gathered and kept in liquid nitrogen for 15 min after centrifugation by vortex. From control or HOXA11‐AS silenced DU145 cells, metabolites were collected. The computer‐aided Similarity Evaluation System for Chromatographic Fingerprint of TCM (version 2012), SIMCA software (Umetrics, Sweden), and Trace1300 GC–MS analytical equipment from Thermo Scientific were all used for the metabolomics analysis.

### 2.12 Animal studies

The Animal Experimentation Center at Xiangya Medical College provided us with 10 female naked mice. The Xiangya Medical College Ethics Committee gave its approval for the animal experiment. Mice were housed in a germ‐free setting. Under the axillary skin of the naked mice, which were randomly assigned to the control group (si‐con, *n* = 5) and the HOXA11‐AS knockdown group (si‐HOXA11‐AS, *n* = 5), cell suspensions of adjusted concentrations were injected. After 16 days of inoculation, tumour samples were acquired after killing nude mice, and the volume of tumours in each group was measured.

### 2.13 Histological analysis

We fixed tumour tissues in 4% paraformaldehyde for the night. Samples were dehydrated, and then embedded in paraffin, thinly sectioned and haematoxylin and eosin stained. The samples were treated overnight after being exposed to the primary antibody Ki67 during immunohistochemistry as per the manufacturer's instructions. Incubate at 37°C for 30 min with two pits. And stained Ki67 positive cells with DAB (3,3′‐diaminobenzidine) in brown colour.

### 2.14 Statistical analysis

SPSS, GraphPad Prism 8 (GraphPad Software, USA), and R were used to conduct the statistical analyses. One‐way analysis of variance (ANOVA), multiple comparisons, and *t*‐tests were used to evaluate statistical significance. Statistics were judged significant at *p* < 0.05.

## 3 RESULTS

### 3.1 LncRNA HOXA11‐AS expression was associated with the clinical performance of PCa

**FIGURE 1:**
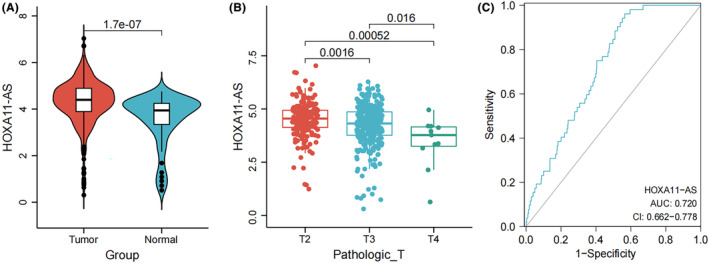
LncRNA HOXA11‐AS expression patterns. (A) Violin plot of lncRNA HOXA11‐AS transcriptome expression levels between samples obtained from healthy individuals and PCa patients. (B) Box line plot of HOXA11‐AS expression at different pathologic T stages. (C) Diagnostic ROC curve of HOXA11‐AS expression.

We looked at HOXA11‐AS expression across a range of tissues and clinical stages. Between PCa and normal tissues, HOXA11‐A was significantly different (*p* = 1.7e‐07) (Figure 1A). To explore the association between the pathologic T stage and HOXA11‐AS, Figure 1B shows the outcomes of our analysis of HOXA11‐AS expression levels in various pathologic T stages. The expression levels of HOXA11‐AS were markedly inversely linked with the Pathologic T stage and considerably dropped as the pathologic T stage increased. The ROC curve showed that HOXA11‐AS could effectively determine tumour versus normal samples (Figure 1C). HOXA11‐AS is up‐regulated in tumour tissues with determined by its differences at the transcriptome level. However, the T grade is a clinical indicator rather than a transcriptomic indicator.

### 3.2 HOXA11‐AS was correlated with EMT and glycolysis

**FIGURE 2:**
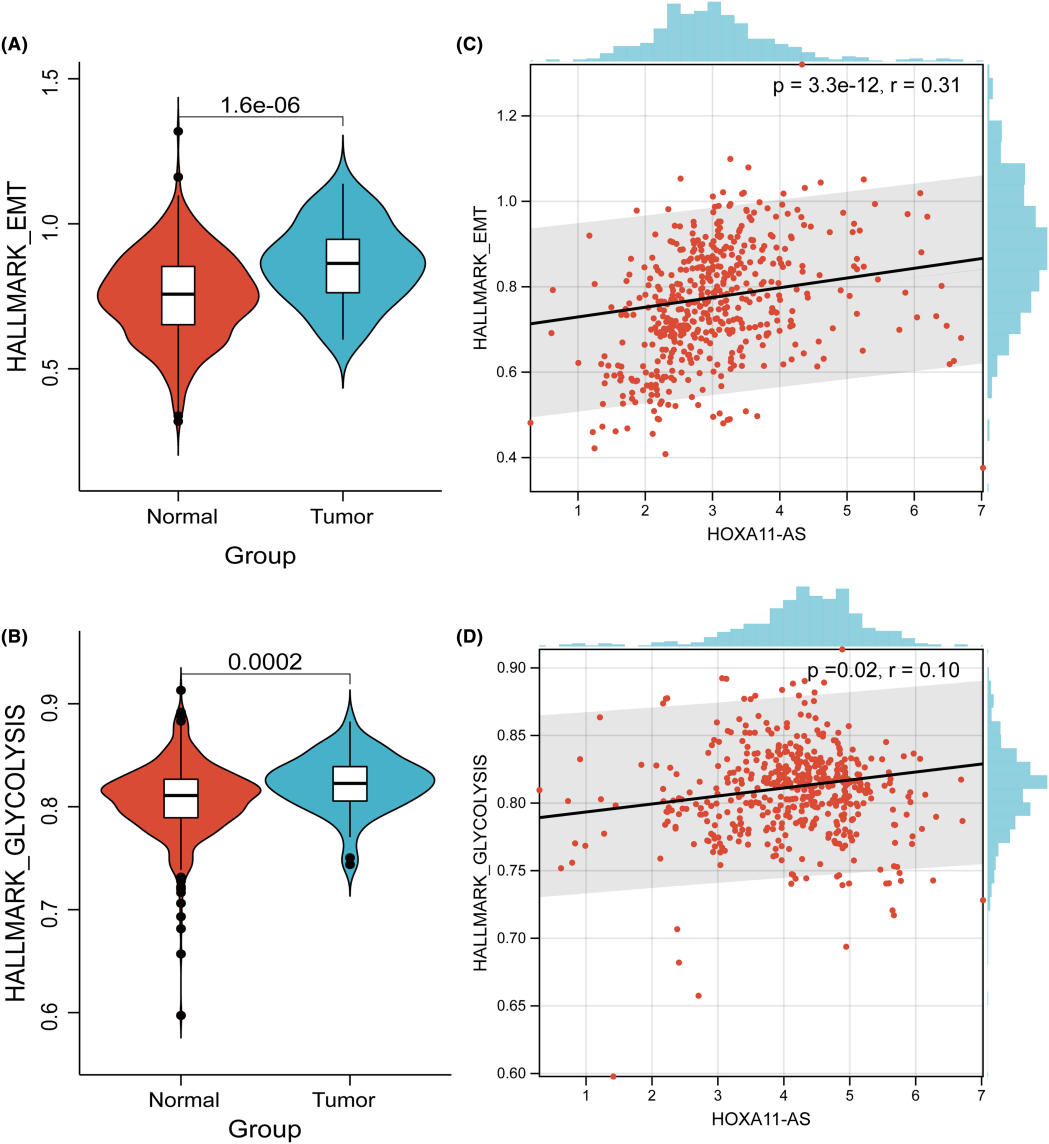
Analysis of correlation of HOXA11‐AS levels with EMT and glycolysis. (A, B) Violin plots of enrichment‐related differences in EMT and glycolysis scores between healthy individuals and PCa patients. (C, D) Scatter plots of correlation of HOXA11‐AS levels with EMT and glycolysis.

EMT and glycolysis scores were calculated separately in each sample through the ssGSEA algorithm. We found significant differences in EMT (*p* = 1.6e‐06) and glycolysis (*p* = 0.0002) scores between PCa and normal tissue (Figure 2A,B). EMT and glycolysis scores were significantly upregulated in PCa tissues compared to normal tissues. To explore the association between HOXA11‐AS and EMT and glycolysis, we calculated the correlation between HOXA11‐AS and EMT and glycolysis by cor function, where we found that HOXA11‐AS was significantly correlated with EMT (Figure 2C; *p* = 3.3e‐12, *r* = 0.31) and glycolysis (Figure 2D; *p* = 0.02, *r* = 0.10) were significantly correlated.

### 3.3 Downstream key genes of HOXA11‐AS demonstrated good clinical diagnostic performance

**FIGURE 3:**
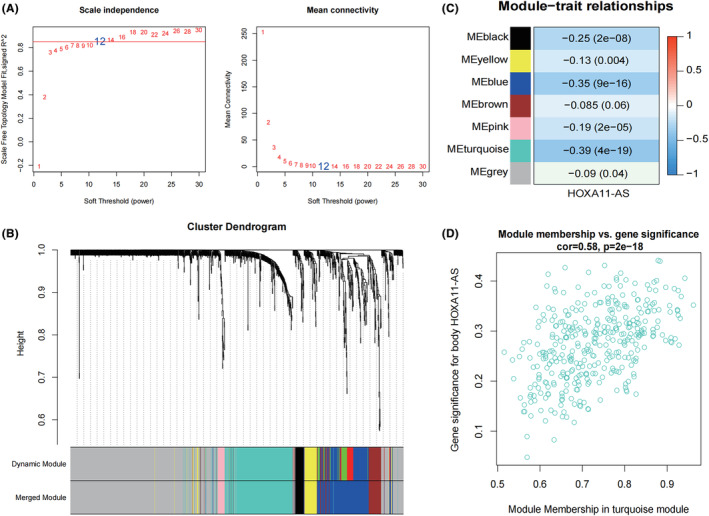
WGCNA analysis. (A) A plot of the power of the adjacency matrix weight parameters. The vertical axis shows the squared value of the correlation coefficient between log(k) and log(p(k)) in the relevant network, while the horizontal axis depicts the power of the weight parameters. Increased squared correlation coefficient values show that the network is getting closer to a network‐free scale distribution. Right: gene average connectivity is shown schematically for various adjacency matrix weight parameters and power parameters. The red line denotes the standard line, where the square of the correlation coefficient results in a value of 0.85. Red lines for the values of the adjacency matrix weight parameters and power parameters represent the average degree of connectivity between network nodes. (B) Module partitioning tree diagram. Different modules are shown in different colours. (C) A heat map of the correlation of each module with clinical phenotypes. (D) Scatter plot of the correlation of turquoise modules with HOXA11‐AS.

WGCNA analysis is based on 1289 concatenated gene expression matrices, with HOXA11‐AS expression as the clinical phenotype. We have to investigate the neighbourhood matrix weight parameter power values. When the correlation coefficient's squared value initially hit 0.85 (red line), we decided on the value of power to be 12 (Figure 3A). The highly correlated genes were clustered into modules. The 1289 concatenated genes were divided into seven modules (Figure 3B). Subsequently, Figure 3C displays the results of calculating the relationship between each module and the clinical phenotype. We focused on the turquoise module with the strongest correlation with HOXA11‐AS, and subsequent investigation showed that this module had a substantial correlation with HOXA11‐AS (Figure 3D). Therefore, the 272 genes contained in the turquoise module were used as module genes for subsequent analyses. Using the cor function, we calculated the Pearson Correlation Coefficient (PCC) of the expression levels between HOXA11‐AS and 1289 genes. Genes with absolute PCC > 0.35 and significant *p* < 0.05 were selected and compared with the above turquoise module to take the intersecting genes as key genes for subsequent analysis.

**FIGURE 4:**
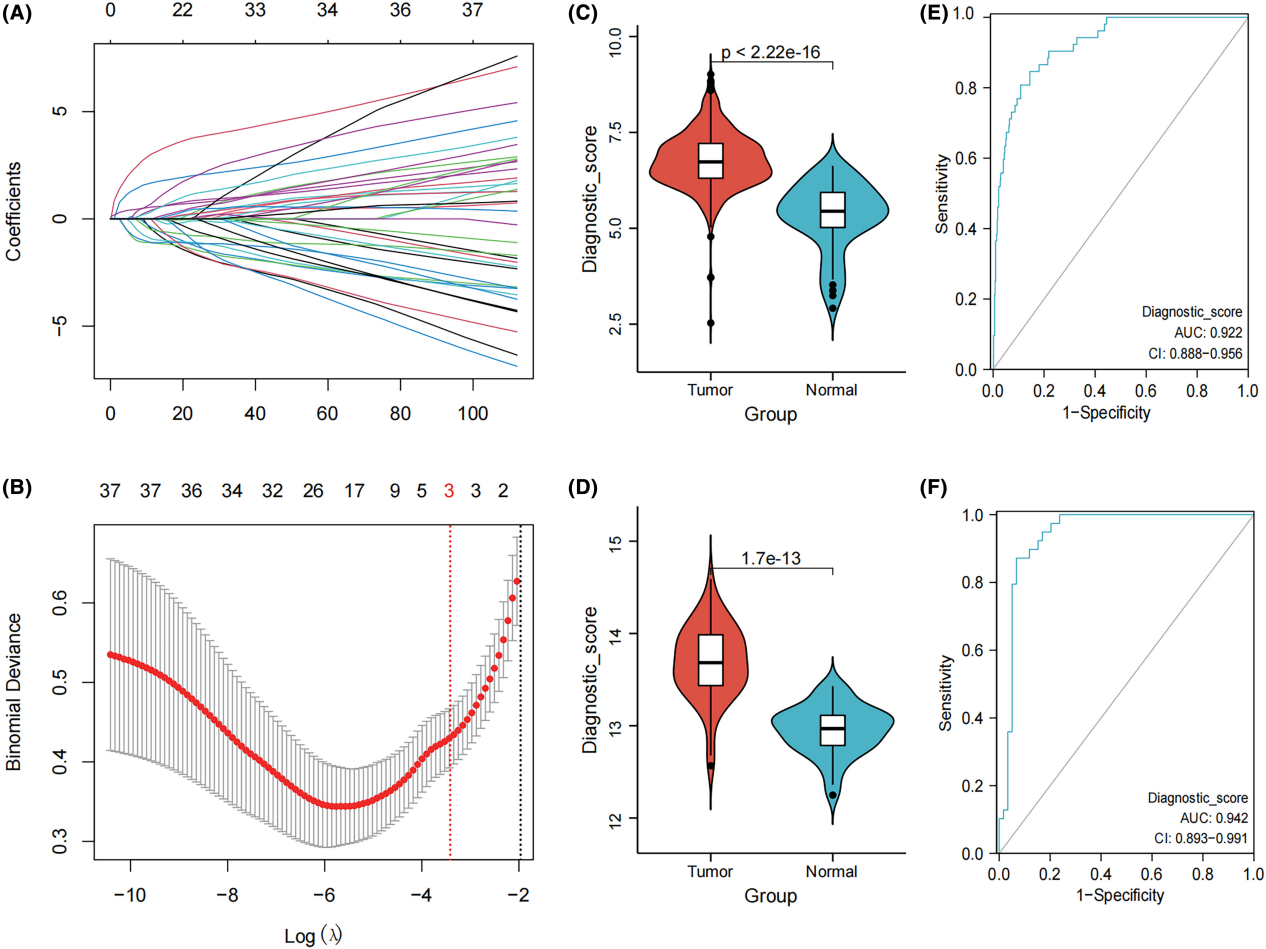
Diagnostic score construction and validation. (A) Distribution of LASSO coefficients. (B) Likelihood deviation of the LASSO coefficient distribution; two vertical dashed lines represent the lambda.min (left red line) and lambda.1se (right black line). (C, D) Violin plots of differences in diagnostic scores across samples. (E, F) ROC curves for diagnostic scores.

We identified 37 significant genes using one‐way logistic regression. Three optimally characterized genes, B3GALT6, IDUA and SPDEF, were then obtained by LASSO logistic modelling (Figure 4A,B). Diagnostic_score was constructed using LASSO regression prognostic coefficients and expression values in the TCGA dataset. Diagnostic_score = 1.0468*EXP_B3GALT6_ + 0.2783*EXP_IDUA_ + 0.2204*EXP_SPDEF_.

It was found that the diagnostic scores in both the training and validation sets were significantly different in the tumour and normal samples (Figure 4C,D). And the ROC curves confirmed the good diagnostic performance of Diagnostic_score with AUC values above 0.9 (Figure 4E,F).

### 3.4 PCa cells exhibit enhanced proliferate, migrate capacity and undergo EMT when HOXA11‐AS is overexpressed

**FIGURE 5:**
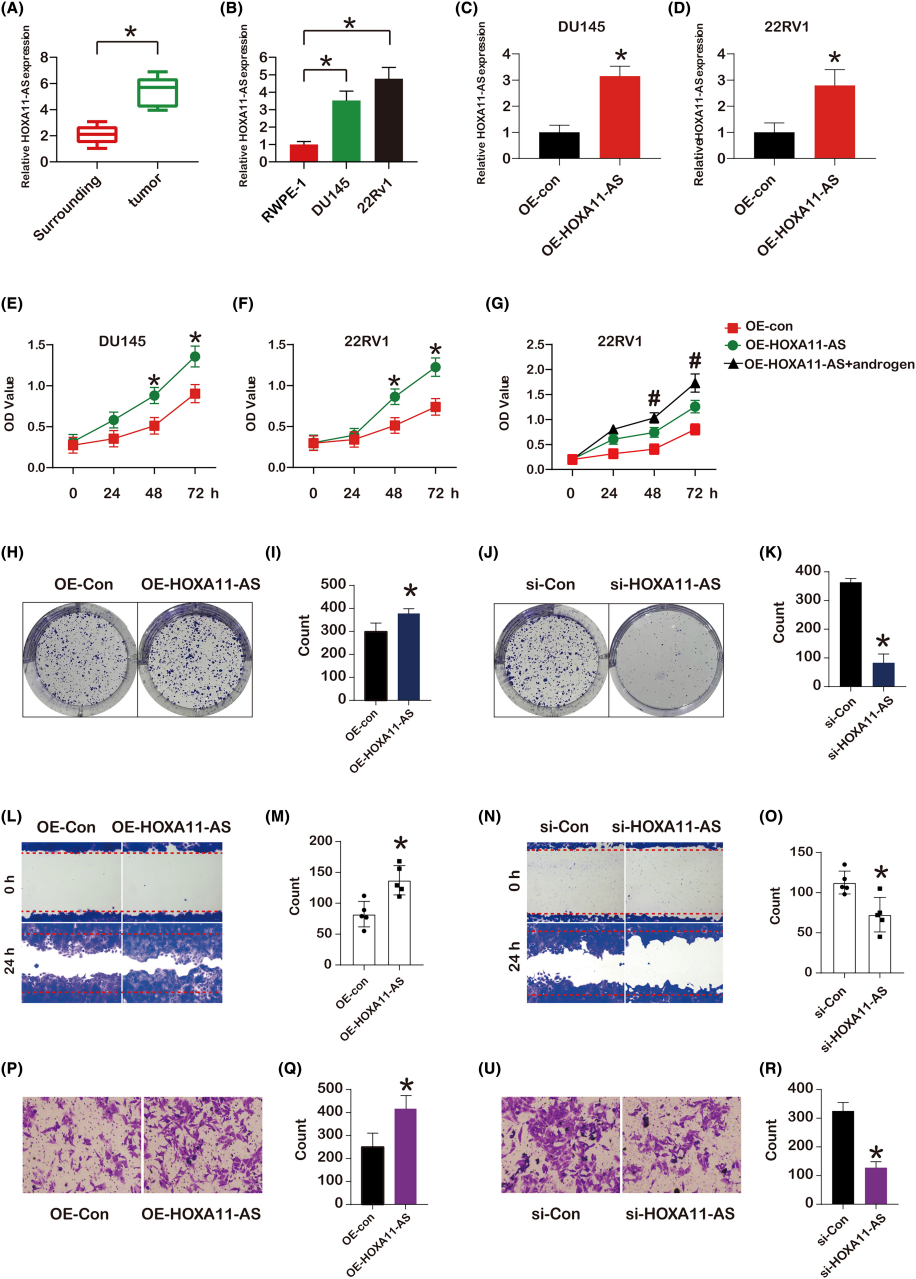
LncRNA HOXA11‐AS promote prostate cancer cell proliferation, migration. (A) LncRNA HOXA11‐AS expression patterns in tumour and surrounding tissues (*n* = 3), (B) the expression patterns of LncRNA HOXA11‐AS in tumour cells (DU145, 22Rv1) and prostate epithelial cells (RWPE‐1) (*n* = 3). (C, D) the identification of HOXA11‐AS expression in DU145 and 22RV1 cells with OE of HOXA11‐AS (*n* = 3). (E, F) OE HOXA11‐AS stimulate the proliferation of DU145 and 22RV1 cells in 72 h; (g) the androgen further up‐regulated the cell proliferation in 22RV1 cells treated with OE‐HOXA11‐AS compare to the oe‐HOXA11‐AS group without androgen. (*) *p* < 0.05 against 0 h denotes a significant difference (*n* = 3). (H, I) Colony formation of DU145 cells treated with OE‐HOXA11‐AS (*n* = 3). (J, K) Colony formation of DU145 cells treated with si‐HOXA11‐AS (*n* = 3). (L, M) the scratch of DU145 cells treated with OE‐HOXA11‐AS (*n* = 3). (N, O) the scratch of DU145 cells treated with si‐HOXA11‐AS (*n* = 3). (P, Q) the invasion of DU145 cells treated with OE‐HOXA11‐AS (*n* = 3). (R, S) the invasion of DU145 cells treated with si‐HOXA11‐AS (*n* = 3).

**FIGURE 6:**
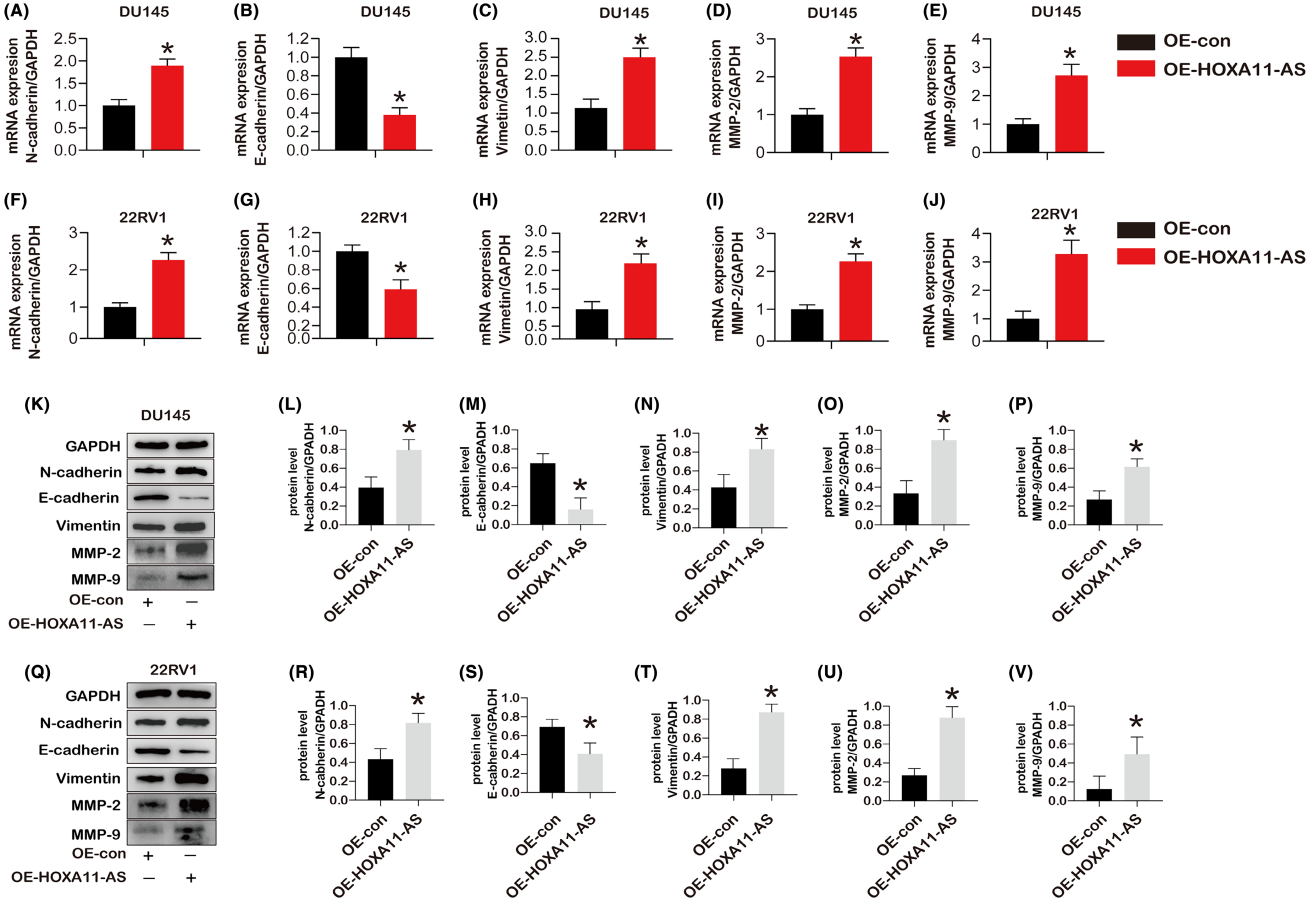
The mRNA and protein expression of EMT markers (E‐cadherin, N‐cadherin, and vimentin) and migration markers (MMP‐2 and MMP‐9) in cells after treatment with OE‐HOXA11‐AS. (A–E) the gene expression of E‐cadherin, N‐cadherin, vimentin, MMP‐2, and MMP‐9 in DU145 cells with HOXA11‐AS over expression treatment for 24 h (*n* = 3). (F–J) the gene expression of E‐cadherin, N‐cadherin, vimentin, MMP‐2, and MMP‐9 in 22RV1 cells with HOXA11‐AS over expression treatment for 24 h (*n* = 3). (K–P) the protein expression of E‐cadherin, N‐cadherin, vimentin, MMP‐2, and MMP‐9 in DU145 cells with HOXA11‐AS over expression treatment for 24 h (*n* = 3). (Q–V) the protein expression of E‐cadherin, N‐cadherin, vimentin, MMP‐2, and MMP‐9 in 22RV1 cells with HOXA11‐AS over expression treatment for 24 h (*n* = 3). (*) *p* < 0.05 versus OE‐con (control) indicates significant difference.

Using qPCR, HOXA11‐AS mRNA expression in surrounding and tumour tissues next to malignancy in PCa patients was studied. Compared to surrounding tissues, tumour tissues had higher levels of HOXA11‐AS expression (Figure 5A). Then we studied the HOXA11‐AS expression in PCa cells (DU145 and 22RV1). DU145 and 22RV1 cells shown higher HOXA11‐AS expression than RWPE‐1 (Figure 5B). After overexpression, HOXA11‐AS upregulation in DU145 and 22RV1 cells was investigated. Additionally, for both PCa cells, comparing the overexpression group to the control, HOXA11‐AS had a higher expression (Figure 5C,D). The impact of HOXA11‐AS overexpression on cell proliferation was the second thing we looked at. The outcomes demonstrated that HOXA11‐AS overexpression increased cell proliferation in DU145 and 22RV1 cells (Figure 5E,F). Furthermore, androgen further up‐regulated the cell proliferation in 22RV1 cells treated with OE‐HOXA11‐AS compare to the oe‐HOXA11‐AS group without androgen (Figure 5G). Next, we found that OE‐HOXA11‐AS led to an increase of colony formation in DU145 (Figure 5H,I). Accordingly, the si‐HOXA11‐AS led to a decrease of colony formation in DU145 (Figure 5J,K). We performed a wound healing assay to determine how HOXA11‐AS regulated PCa cell migration. HOXA11‐AS overexpression increased the migration of DU145 cells compared to the oe‐control, but si‐HOXA11‐AS decreased the migration of DU145 cells compared to the si‐control (Figure 5L–O). Moreover, the trans‐well assay was used to explore the effects of the HOXA11‐AS on the PCa cell invasion. The results showed that oe‐HOXA11‐AS promoted the invasion of the DU145 cells, but the si‐HOXA11‐AS inhibited the invasion of the DU145 cells (Figure 5P–S). EMT is an essential factor in the invasive metastasis of PCa cells. Therefore, we next determine whether EMT markers were altered by q‐PCR and western blot. The genes expression of E‐cadherin, N‐ cadherin, vimentin, MMP‐2 and MMP‐9 in DU145 cells (Figure 6A–E) and 22RV1 cells (Figure 6F–J) was observed. The protein level of N‐cadherin, vimentin, MMP‐2 and MMP‐9 was significantly increased, and the protein expression of E‐cadherin and were decreased in both DU145 cells (Figure 6K–P) and 22RV1 cells (Figure 6Q–V) oe‐HOXA11‐AS compare to the oe‐control. These findings imply that overexpression of HOXA11‐AS promotes PCa cell migration and EMT process.

### 3.5 HOXA11‐AS knockdown inhibits prostate cancer cell migration and proliferation

**FIGURE 7:**
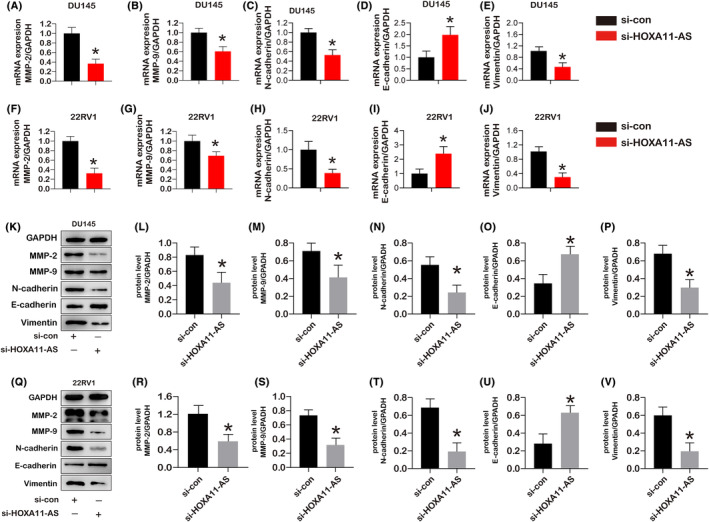
The mRNA and protein expression of EMT markers (E‐cadherin, N‐cadherin, and vimentin) and migration markers (MMP‐2 and MMP‐9) in cells after treatment with si‐HOXA11‐AS. (A–E) the gene expression of E‐cadherin, N‐cadherin, vimentin, MMP‐2, and MMP‐9 in DU145 cells after treatment with si‐HOXA11‐AS for 24 h (*n* = 3). (F–J) The gene expression of E‐cadherin, N‐cadherin, vimentin, MMP‐2, and MMP‐9 in 22RV1 cells after treatment with si‐HOXA11‐AS for 24 h (*n* = 3). (K–P) the protein expression of E‐cadherin, N‐cadherin, vimentin, MMP‐2, and MMP‐9 in DU145 cells after treatment with si‐HOXA11‐AS for 24 h (*n* = 3). (Q–V) the protein expression of E‐cadherin, N‐cadherin, vimentin, MMP‐2, and MMP‐9 in 22RV1 cells after treatment with si‐HOXA11‐AS for 24 h (*n* = 3). (*) *p* < 0.05 versus OE‐con (control) indicates significant difference.

We employed siRNA to suppress HOXA11‐AS expression to further examine the role of HOXA11AS in PCa migration. Therefore, we also determine whether EMT markers were altered in si‐HOXA11‐AS by q‐PCR and western blot. The genes expression of E‐cadherin, N‐ cadherin, vimentin, MMP‐2 and MMP‐9 in DU145 cells (Figure 7A–E) and 22RV1 cells (Figure 7F–J) was observed. The protein level of N‐cadherin, vimentin, MMP‐2 and MMP‐9 was significantly decreased, and the gene and protein expression of E‐cadherin were increased in si‐ HOXA11‐AS compare to the si‐control in both DU145 cells (Figure 7K–P) and 22RV1 cells (Figure 7Q–V). These findings imply that si‐HOXA11‐AS inhibits the PCa cell migration and EMT process.

### 3.6 LncRNA HOXA11‐AS regulates glycolytic metabolism in PCa cells

**FIGURE 8:**
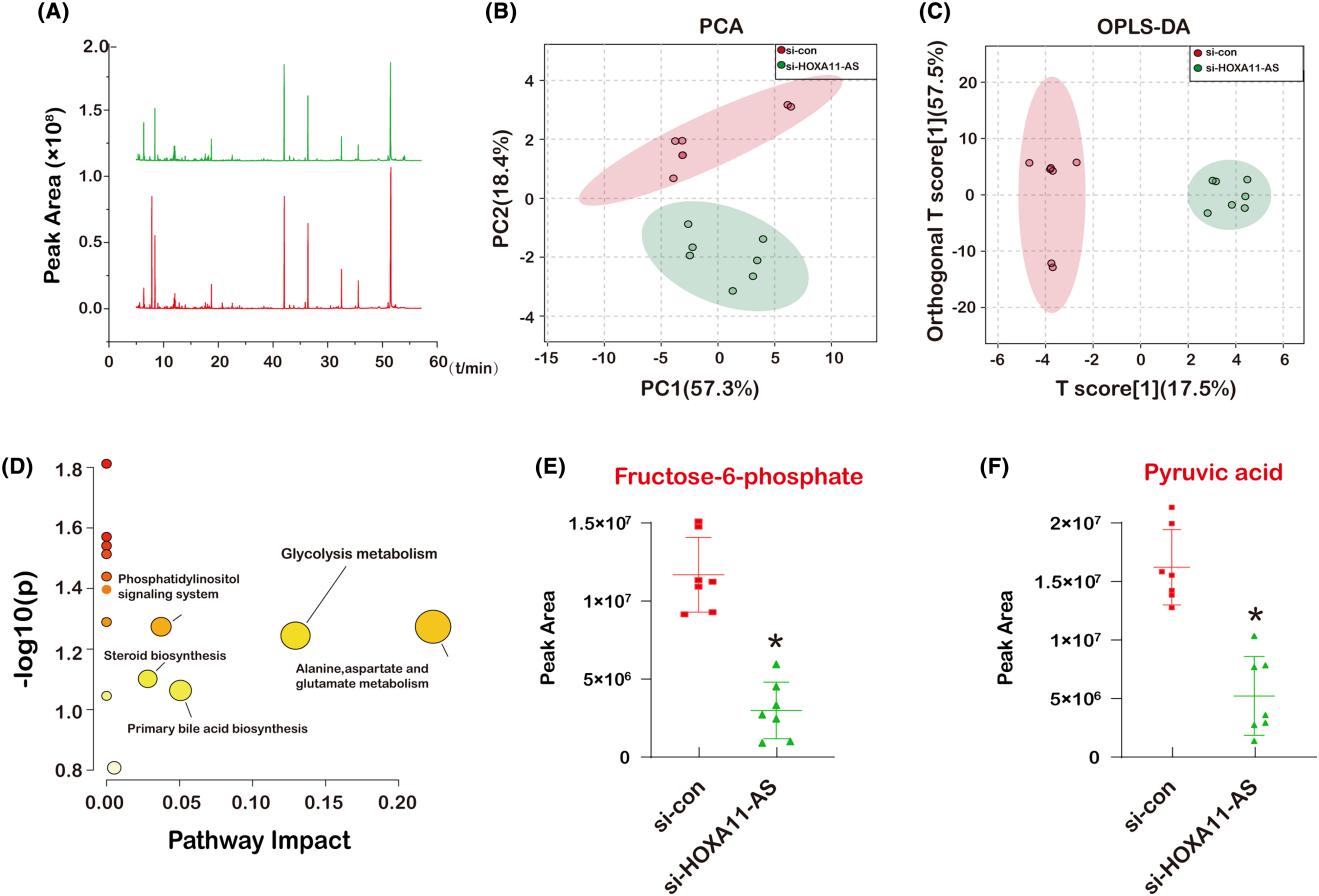
LncRNA HOXA11‐AS regulates the Glycolytic metabolism in prostate cancer cell. (A) TIC (Total Ion Chromatogram) of GC–MS of the cells with si‐HOXA11‐AS treatment. (B) PCA analysis of si‐con (control) and si‐HOXA11‐AS groups, which represent that the samples of both groups are closely clustered with each other. (C) OPLS‐DA analysis of si‐con (control) and si‐HOXA11‐AS groups displaying how the samples in the training group were clustered. (D) The plots compare the effects of significant metabolites on the metabolic pathways between the control and si‐HOXA11‐AS groups and calculated metabolic pathways as a function of −log(p) (y‐axis). The circles' colours, yellow for low enrichment significance and red for high enrichment importance, indicated the degree of enrichment. The size of the circles represents how much of an influence the pathway has. (E, F) Peak area of selected differential metabolites of the DU145 cells in si‐con and si‐HOXA11‐AS groups. Data are presented as mean SD, where *n* is 7. *p < 0.05 by one‐way ANOVA.

To learn more about how PCa cells' glycolytic metabolism is impacted by the lncRNA HOXA11‐AS, we used GC–MS to analyse changes in the metabolic components of DU145 cells in the control and si‐HOXA11‐AS groups (Figure 8A). By comparing the metabolic changes in the control and si‐HOXA11‐AS groups by PCA analysis, we separate the control and si‐HOXA11‐AS groups differing significantly according to the first predicted principal component (Figure 8B). In addition, we compared metabolic changes in the control and si‐HOXA11‐AS groups by OPLS‐DA analysis. Applying an orthogonal filter in OPLS‐DA has the advantage of targeting and separating metabolic changes corresponding to specific factors (e.g. pathological effects), even if the magnitude affected by the pathological state is small. OPLS‐DA score with linear classifier boundaries shows a clear separation between the si‐con (control) and si‐HOXA11‐AS groups (Figure 8C). Metabo analyst pathways were analysed for metabolic gene pathways in the control and si‐HOXA11‐AS groups. Gene analysis revealed metabolic changes mainly associated with glycolysis, phosphatidylinositol signalling pathway and aspartate alanine and glutamate metabolic pathway metabolism (Figure 8D). We analysed the differences in fructose‐6‐phosphate and pyruvate acid in the two groups of cells independently to further compare the variations in glycolytic metabolites between the control and si‐HOXA11‐AS groups. Fructose‐6‐phosphate and pyruvate acid levels in the si‐HOXA11‐AS group were considerably lower, according to the findings (Figure 8E,F).

### 3.7 The silence of HOXA11‐AS significantly inhibited tumour growth in vivo

**FIGURE 9:**
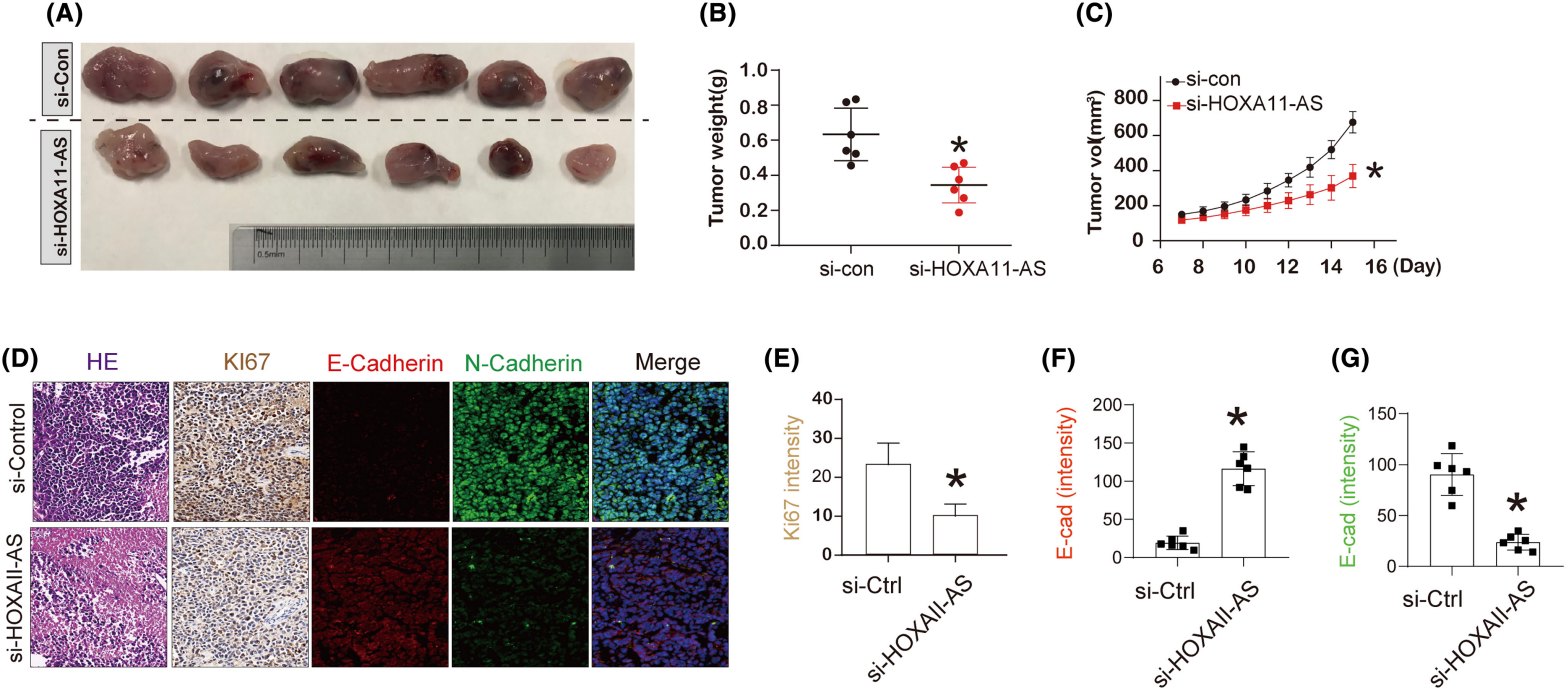
Tumour development is slowed in vivo when lncRNA HOXA11‐AS is silenced. (A) tumours isolated from nude mice, and their macro appearance were shown. (B) Tumour weight of isolated tumours with si‐con (control) and si‐HOXA11‐AS DU145 prostate cancer cells xenograft. (C) Tumour volume detection of different groups in 16 days. (D) Haematoxylin and eosin staining and IHC of KI67; the immunofluorescence staining of E‐cadherin and N‐cadherin on tumour samples. (E–G) Quantitative analysis of KI67 and E‐cadherin and N‐cadherin data from (D). *p < 0.05 versus si‐con indicates significant difference.

We injected DU145 cells as xenografts into nude mice to see if HOXA11‐AS knockdown could prevent the growth of tumours in vivo. Tumours were isolated from the nude mice 16 days later (Figure 9A). We assessed the tumour weight and volume in the si‐HOXA11‐AS group of mice and discovered a considerable reduction (Figure 9B,C). To further analyse the antitumor effect of si‐HOXA11‐AS, we performed haematoxylin and eosin staining, immunohistochemistry staining with Ki67 and immunofluorescence staining with E‐cadherin and N‐cadherin on tumour samples. Compared with the negative control group, the number of cells in the si‐HOXA11‐AS group was significantly reduced (Figure 9D). Further, immunohistochemical results showed that the expression of the proliferation marker protein Ki67 was decreased in si‐HOXA11‐AS group compare with the si‐control group (Figure 9D,E). The immunofluorescence results showed that si‐HOXA11‐AS significantly promoted the expression of N‐cadherin, but inhibited the E‐cadherin expression compare to the si‐control (Figure 9D,F,G). All animal experiments showed si‐HOXA11‐AS suppressed tumour growth in vivo.

## 4 DISCUSSION

In this study, we found that patients exhibiting HOXA11‐AS overexpression had a poorer prognosis for survival and that lncRNA HOXA11‐AS expression levels were higher in PCa tissues. It is well known that HOXA11‐AS is expressed in PCa and its functions have been verified. Key genes downstream of HOXA11‐AS exhibited good clinical diagnostic properties for PCa. HOXA11‐AS overexpression increased PCa cell proliferation, migration, and EMT, while HOXA11‐AS silencing had contrasting effects on PCa cells. In addition, HOXA11‐AS silencing downregulated multiple metabolites through the glycolytic pathway. In vivo experiments showed that HOXA11‐AS silencing had a significant inhibitory effect on tumour growth in vivo.

HOXA11‐AS may be a viable biomarker for the early diagnosis and prognostic assessment of cancer patients.^6^ HOXA11‐AS lncRNA has been shown to perform an oncogenic role and is overexpressed in various malignancies.^20^, ^21^, ^22^ According to a previous study, HOXA11‐AS expression was markedly upregulated in hypopharyngeal squamous cell carcinoma and was positively correlated with lymph node metastasis.^23^ According to the TCGA database, HOXA11‐AS was significantly expressed in lung adenocarcinoma and squamous cell carcinoma. When used as a diagnostic paradigm, the ROC curve showed that the area under the curve (AUC) values for HOXA11‐AS were 0.727 and 0.933 for patients with lung adenocarcinoma and squamous cell carcinoma, respectively.^24^ Chen et al. found that HOXA11‐AS expression was significantly higher in ovarian cancer cells than in healthy cells, which is consistent with our findings.^25^ Our findings showed that HOXA11‐AS expression levels were inversely linked with the pathologic T stage and notably decreased with an increase in the pathologic T stage. ROC curves show that HOXA11‐AS can effectively distinguish prostate tumour samples from healthy tissue samples.

A substantial link was identified between HOXA11‐AS and EMT and glycolysis via the integration of bioinformatic analysis techniques. In contrast, the EMT‐directed invasive growth of tumour cells was closely associated with enhanced aerobic glycolysis.^26^ Epithelial cells acquired the capacity to migrate and invade through EMT, while losing their cell polarity and intercellular adhesive ability.^1^, ^27^, ^28^ EMT is linked to a complex metabolic reprogramming process controlled by transcription factors specific to EMT that support higher energy needs for growth and motility in challenging environments.^29^ The silencing of metabolic genes activates EMT, demonstrating that EMT and glycolytic reprogramming are intertwined.^30^ Cancer cells do not produce much ATP via aerobic glycolysis. Thus, more glucose is consumed, and lactate is produced.^31^ This changed metabolic profile is associated with tumorigenesis and a poor prognosis for cancer patients.^26^ The molecular complexity of cancer metastasis can be understood comprehensively by understanding the mechanisms of EMT and glycolysis.

Our experimental findings demonstrated that the downregulation of HOXA11‐AS reduced glycolytic metabolism. HOXA11‐AS promotes an increase in the glucose transporter protein levels in the glycolytic pathway, which enhances tumour proliferation and migration.^32^ The PI3K/Akt pathway is activated, which facilitates the EMT process by encouraging glycolysis and glucose uptake.^33^ Metabolic studies have reported that HOXA11‐AS knockdown suppressed the glycolytic process.^34^ The knockdown of HOXA11‐AS reduced the NAD coenzyme levels, inhibiting cell proliferation, metastasis, and EMT, and increased apoptosis levels.^35^ The same was observed in our experiments. We also found that metabolic pathways, especially the glycolytic pathway, were altered in PCa cells after HOXA11‐AS silencing. Both fructose‐6‐phosphate and pyruvate acid levels were significantly reduced after HOXA11‐AS silencing. Experiments carried out in vitro demonstrated that HOXA11‐AS knockdown prevented EMT progression and slowed tumour cell migration and proliferation.^12^ This suggests that HOXA11‐AS can reduce the metabolism of tumour cells by suppressing EMTs and glycolytic processes, thereby inhibiting tumour cell growth.

HOXA11‐AS overexpression enhances PCa cell invasion and migration. Cell invasion, proliferation and migration were facilitated in oral squamous cells through HOXA11‐AS overexpression.^34^ HOXA11‐AS overexpression promoted tumour invasion, cell migration and proliferation in vitro, while HOXA11‐AS knockdown inhibited these processes. An increased level of EMTs accompanies these adverse effects.^12^ In hypopharyngeal squamous cell carcinoma, HOXA11‐AS knockdown was found to reduce tumour stemness and EMT.^36^ In addition, HOXA11‐AS might promote metastasis in colorectal cancer by facilitating EMTs.^37^ Our findings have shown that HOXA11‐AS silencing notably reduced MMP2 and MMP9 production in PCa cells, indicating that the overexpression of the lncRNA HOXA11‐AS might encourage PCa cell migration by promoting EMT.

The tumorigenic capacity of PCa cells was significantly reduced because of the silencing of HOXA11‐AS, which is oncogenic in most types of cancer. In hepatocellular carcinoma, the reduced expression levels of stem cell‐related markers showed that HOXA11‐AS overexpression reduced the self‐renewal, proliferation, migration and tumorigenic capacity of HCC stem cells in vivo.^7^ In addition, HOXA11‐AS RNAi induces cell cycle arrest in non‐small cell lung cancer to stop NSCLC cells from proliferating, migrating, invading, and producing tumours. It also triggers apoptosis.^38^ This shows that in vivo tumour development was stimulated by HOXA11‐AS.

## 5 CONCLUSION

Overall, up‐ and down‐regulation of HOXA11‐AS promoted and inhibited the proliferation and migration of PCa cells, respectively. We also found that silencing of HOXA11‐AS resulted in the downregulation of several metabolites of the glycolytic pathway. In addition, silencing of HOXA11‐AS significantly inhibited tumour growth in vivo. In summary, silencing HOXA11‐AS can inhibit PCa by regulating glucose metabolism and is expected to provide new ideas for targeted therapy for PCa.

## AUTHOR CONTRIBUTIONS

**Jiankang Zhang:** Data curation (equal); writing – original draft (equal). **Sailian Li:** Formal analysis (equal); software (equal). **Mengyu Zhang:** Data curation (equal); investigation (equal). **Zhenting Wang:** Methodology (equal); visualization (equal). **Zengshu Xing:** Project administration (equal); supervision (equal); writing – review and editing (equal).

## FUNDING INFORMATION

The Hainan Provincial Natural Science Foundation of China provided funding for this project. (820RC784).

## CONFLICT OF INTEREST STATEMENT

There is no conflict of interest reported by the authors.

## Data Availability

The data that support the findings of this study are available from the corresponding author upon reasonable request.
